# The banded colour patterns of sea snakes discourage attack by predatory fishes, enabling Batesian mimicry by harmless species

**DOI:** 10.1098/rspb.2022.1759

**Published:** 2022-11-30

**Authors:** Claire Goiran, Terri Shine, Richard Shine

**Affiliations:** ^1^ LabEx Corail & ISEA, Université de la Nouvelle-Calédonie, BP R4, 98851 Nouméa cedex, New Caledonia; ^2^ School of Natural Sciences, Macquarie University, New South Wales 2109, Australia

**Keywords:** elapidae, hydrophiinae, laticaudinae, mullerian mimicry, predator-prey

## Abstract

The evolution of bright ‘warning’ colours in nontoxic animals often is attributed to mimicry of toxic species, but empirical tests of that hypothesis must overcome the logistical challenge of quantifying differential rates of predation in nature. Populations of a harmless sea snake species (*Emydocephalus annulatus*) in New Caledonia exhibit colour polymorphism, with around 20% of individuals banded rather than melanic. Stability in that proportion over 20 years has been attributed to Batesian mimicry of deadly snake species by banded morphs of the harmless taxon. This hypothesis requires that banded colours reduce a snake's vulnerability to predation. We tested that idea by pulling flexible snake-shaped models through the water and recording responses by predatory fish. Black and banded lures attracted similar numbers of following fish, but attacks were directed almost exclusively to black lures. Our methods overcome several ambiguities associated with experimental studies on mimicry in terrestrial snakes and support the hypothesis that banded colour patterns reduce a non-venomous marine snake's vulnerability to predation.

## 1. Introduction

The adaptive significance of animal colours and patterns has attracted considerable scientific attention [1]. One intriguing situation involves bright colours and contrasting patterns that do not enhance camouflage, and instead appear to be signals to conspecifics or heterospecifics [2]. Although some cases are driven by sexual selection, the likely benefit in other cases is to advertise the toxicity of the organism displaying those colours. Aposematic (warning) colours that deter attack by predators have evolved in many lineages [3,4]. Given that benefit, a nontoxic species might gain an advantage by adopting those colours—enhancing survival without investing in expensive toxins. That situation can lead to the evolution of mimicry, whereby nontoxic (mimic) species evolve to resemble a sympatric model in appearance and/or behaviour [5,6].

Even in cases where the model-mimic resemblance is extraordinarily precise [7], however, the hypothesis that mimicry has driven that resemblance is difficult to test. Ideally, we need information on selection pressures—for example, how does an individual's degree of resemblance to the putative model affect its probability of survival? Such data are rarely available.

One intensively studied mimicry system in vertebrates involves the evolution of brightly banded (ringed) colour patterns in several lineages of harmless snakes that are sympatric with highly venomous coral snakes (*Micrurus* and related genera) in South and Central America (see reviews by [8,9]). The evidence for a mimetic function relies on concordant geographical variation in coloration of the model versus its putative mimics, reports that predators avoid coral-snake colours, and experimental studies in which physical models (constructed of clay or plasticine) are painted in different colours and deployed in natural habitats. The models are later checked to look for evidence of attacks by avian and mammalian predators [6,10,11]. Several studies have shown that models painted to resemble coral snakes are less likely to be attacked than are models painted in other colours [12,13].

Although these data are compelling, the hypothesis of mimicry-driven evolution of coral-snake colours in nonvenomous snakes remains controversial [9]. For example, resemblance between the model and the putative mimic might reflect convergent selection towards the same phenotypic optimum (perhaps for crypsis), rather than one species evolving to resemble the other. Also, a clay model's colour may affect its detectability rather than predator preference. The force of that argument is diminished by experiments that rendered all models obvious against a plain background [12], but this objection might still be valid if the banded pattern reduces detectability only while a snake is moving (e.g. via flicker-fusion [9,14,15]). Another weakness of the clay-model studies is that marks left by birds and mammals may reflect curiosity or scavenging rather than predation. Given that a dead coral snake poses no threat, an immobile model may not be a convincing replica of a live snake. Predatory coatis (*Nasua narica*) did not avoid live coral snakes, despite aversion of coral-snake-coloured rubber models by the same species [16].

**Figure 1.**
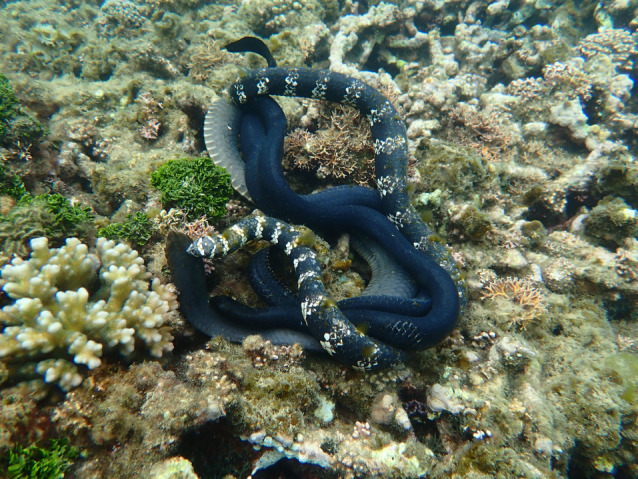
Mating aggregation of turtle-headed seasnakes (*Emydocephalus annulatus*) showing the three colour morphs in our study site. In this photograph, a grey-banded female is being courted by a melanic male and a black-and-white-banded male. Photo by C. Goiran. (Online version in colour.)

Another challenge to testing the ‘coral snake mimicry’ hypothesis is the reliance on interspecific comparisons, because mimic species may differ in numerous ways (other than colour) from the model. A stronger intraspecific test is possible if the putative mimic exhibits a range of colour morphs, with some morphs resembling a toxic model whereas others do not. In that case, we can compare predator responses to different morphs within a single population; and can track relative frequencies of alternative morphs through time to test the prediction that polymorphism is stable (that is, a change in frequency is followed by another change back toward the previous frequencies). Such stability is likely to be due to frequency-dependent selection. We study such a system. In shallow bays around the city of Noumea in the IndoPacific archipelago of New Caledonia, turtle-headed sea snakes (*Emydocephalus annulatus*) exhibit three colour morphs [17] (figure 1). About 80% of individuals are jet-black (melanic) throughout life, about 10% are brightly banded in black-and-white throughout life, and the other 10% are born with indistinct (grey) bands on a black background but transform to melanism as they age [17]. The banded morphs resemble sympatric deadly sea snakes [17]. If this situation arose via mimicry, we would predict that predators avoid banded snakes but attack black ones. We conducted experimental trials to evaluate that prediction.

## 2. Material and methods

### (a) Study sites

We worked at two sites: a small bay (Baie des Citrons) and an island (Ile aux Canards, 22°16′S, 166°26′E) 0.8 km offshore from the first site. Although fishing is legally prohibited at both sites, that prohibition is more stringently enforced on Ile aux Canards than on Baie des Citrons (R.S. 2021, personal observation). As a result, large fishes are more common at Ile aux Canards [18]. Fishes in both sites are habituated to recreational snorkellers but are not artificially fed. At both sites, corals (branching, non-branching and soft) dominate the inshore substrate, giving way to sandy areas at depths greater than 2 m [19,20]. Shallow areas have expanses of coral rubble, with coral bleaching at the Baie des Citrons site.

### (b) Predator fauna

**Figure 2.**
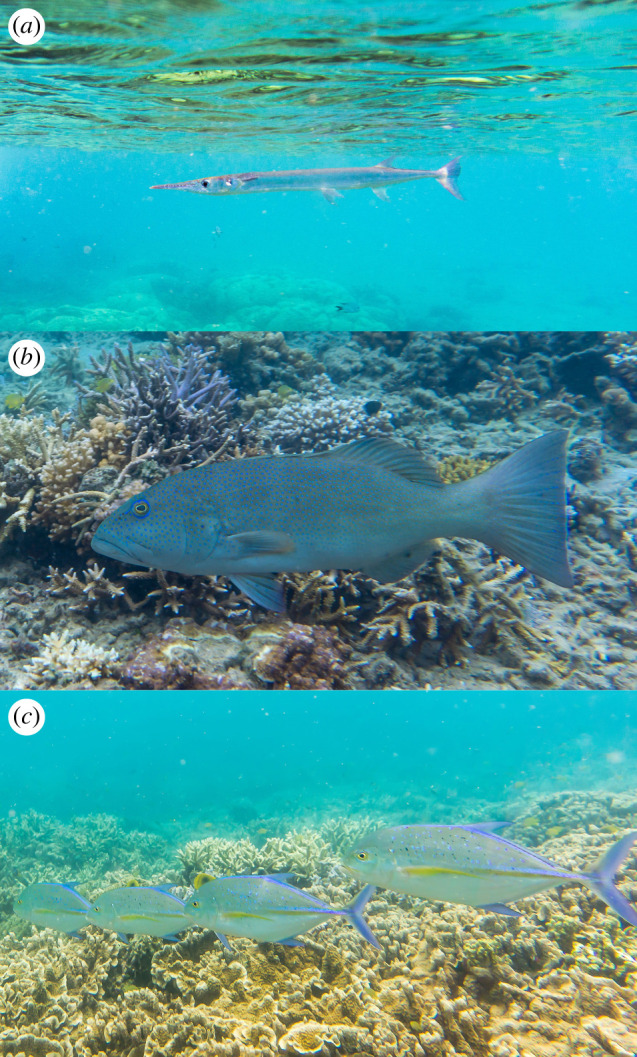
Species of predatory fish present in our study sites that commonly attacked snake-shaped lures. (*a*) Long tom *Tylosurus crocodilus*, (*b*) coral trout *Plectropomus leopardus* and (*c*) blue trevally *Caranx melampygus*. Photographs by T. Shine. (Online version in colour.)

Predators on sea snakes include eagles, ospreys and shorebirds [21], as well as sharks [22,23] and teleost fishes [24,25]. Among teleosts, the most likely predators of sea snakes are benthic fishes such as groupers and coral trout (*Plectropomus*), as well as surface-swimming fishes such as barracuda (*Sphyraena* spp.), trevally (*Caranx* spp.) and needlefish (*Tylosurus*) (figure 2). In New Caledonia, sea snakes are consumed by sharks [26], and we have reports of two melanic *E. annulatus* in the stomachs of yellowtail emperors *Lethrinus atkinsoni* (T. Read 2021, personal communication), and an attack on a juvenile *E. annulatus* by a chocolate grouper *Cephalopholis boenak* (A. Guemas 2021, personal communication; see https://www.inaturalist.org/observations/116240077). Likewise, Heatwole [25] reported predation on Australian sea snakes by other *Lethrinus* spp. and by a grouper (*Epinephalus lanceolatus*). Declines in the abundance of sea snakes in protected areas have been attributed to predation by recovering populations of fishes [19,27].

### (c) Snake fauna

#### (i) Models

**Figure 3.**
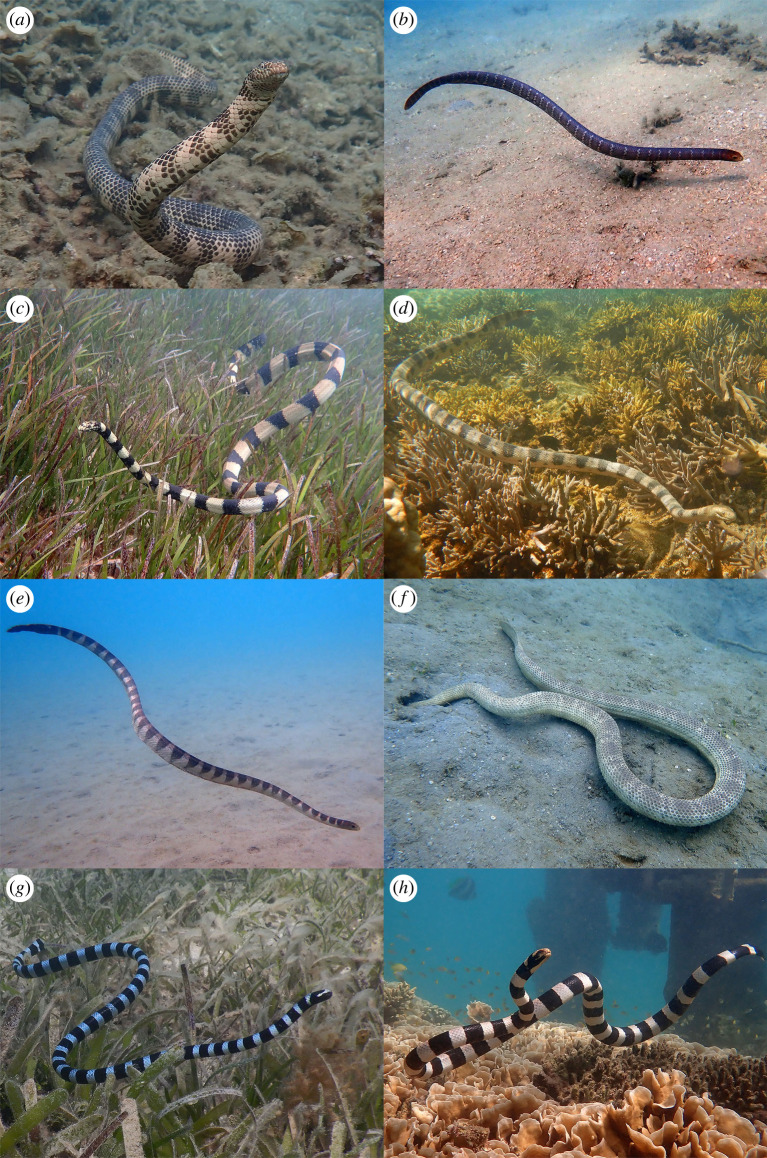
Species of sea snakes present in our study sites. (*a*) *Aipysurus duboisii*, (*b*) *Aipysurus laevis* (juvenile), (*c*) *Hydrophis coggeri*, (*d*) *Hydrophis major*, (*e*) *Hydrophis ornatus*, (*f*) *Hydrophis peronii*, (*g*) *Laticauda laticaudata* and (*h*) *Laticauda saintgironsi*. Photographs by C. Goiran. (Online version in colour.)

The southern part of the great lagoon of New Caledonia contains nine species of dangerously venomous hydrophiine and laticaudine sea snakes. These include two species of *Aipysurus* (*A. duboisii* and *A. laevis*), five species of *Hydrophis* (*H. coggeri*, *H. macdowelli*, *H. major*, *H. peronii*, *H. ornatus*), and two sea kraits (*Laticauda laticaudata*, *L. saintgironsi*) [28,29]. All of these species exhibit moderate to strong banding (figure 3). Bands are brightest in juveniles and fade with age, especially in *A. laevis* [28].

#### (ii) Mimics

The remaining species of hydrophiine, *Emydocephalus annulatus*, is harmless: feeding only on tiny fish eggs, it has lost the potent venom and venom-delivery systems that characterize other sea snakes [24]. This species also is distinctive in exhibiting colour polymorphism: only around 20% of individuals exhibit bands (see above). Our long-term (20-year) mark–recapture studies on these sea snakes [17,30] show that morph frequencies have remained stable. That is, a year with a high proportion of melanic individuals is followed by recruitment of a cohort containing a high proportion of banded individuals, and vice versa for the other colour morphs. Thus, the relative frequencies of the three morphs appear to be maintained by a selective advantage that accrues to whichever morph is less common [17].

What evolutionary forces maintain this colour polymorphism? Melanism enables snakes to excrete pollutants (trace elements) by concentrating toxins in the skin and increasing the frequency with which the skin is sloughed [31]. But why then are some individuals banded? The answer may involve Batesian mimicry of other sea snakes that are brightly banded (resembling the black-and-white morph of *E. annulatus*) or have subtle bands (resembling the grey-banded morph). Under this hypothesis, the relative frequencies of mimics are stable through time because the advantage to mimicry decreases when banding is no longer strongly associated with toxicity. If the banded morphs of *E. annulatus* become common, the deterrent value falls because predatory attacks to banded snakes are less likely to be followed by envenomation of the predator [12,13,17]. Empirically testing the frequency-dependence of any advantage to a specific colour morph is logistically challenging, and would require a system with spatial or temporal variation in morph frequencies. Such variation is minor within Noumea populations of *Emydocephalus annulatus*, because of frequency-dependent selection (see above), so all we can test is the critical assumption that banded snakes are less vulnerable to predation than are unbanded snakes.

### (d) Experimental procedures

**Figure 4.**
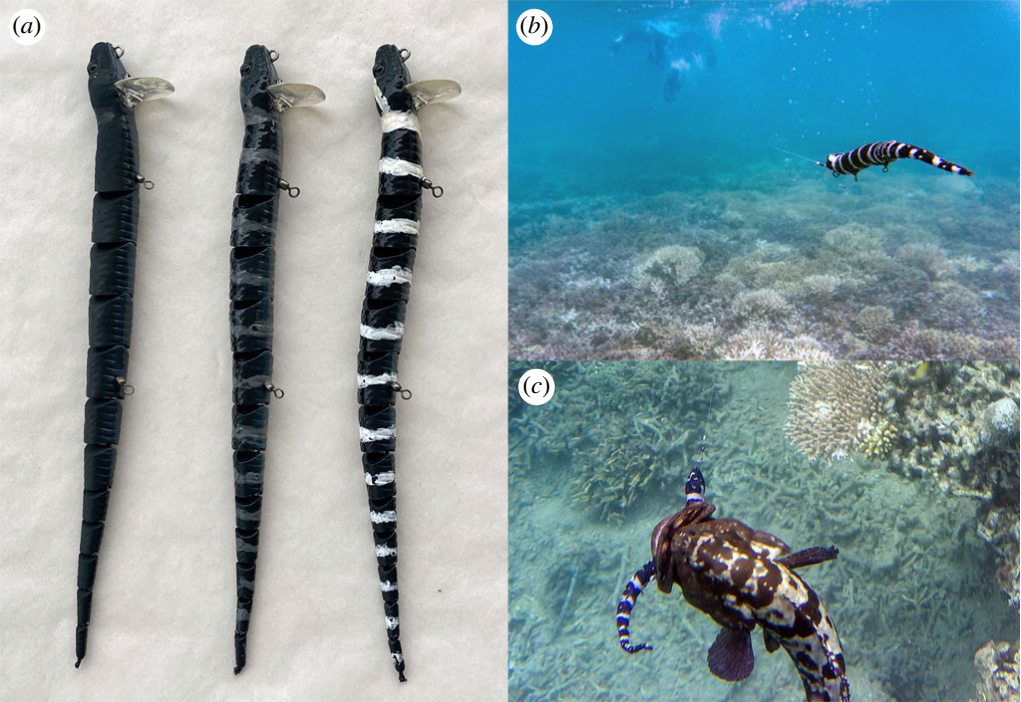
Examples of experimental methods. (*a*) Lures representing the three colour morphs of *Emydocephalus annulatus*, *(b)* snorkeller dragging a lure and (*c*) grouper seizing a lure. Photographs by T. Shine. (Online version in colour.)

Studies on coral snake mimicry have used clay models, which rely upon actively searching avian and mammalian predators treating an immobile model as a potential prey item [9]. By contrast, most of the fishes likely to eat sea snakes are ambush predators such as groupers, that lie in wait for moving prey [32]. As a result, an immobile model would be inappropriate. We pulled fibreglass models of snakes through the water to elicit feeding responses by fishes. These models were commercially made fishing lures (Savage Gear three dimension), 300 mm long (the same length as a newborn *E. annulatus* [30]), with each lure consisting of 12 linked segments to create a sinuous movement that mimics the swimming action of a snake (see https://www.youtube.com/watch?v=PV8R3wwd9xo for video of the lure's action). We removed the hooks and added weights (Storm Suspenstrips) to the ventral surface of the lure to ensure negative buoyancy. We used acrylic craft paint (Born Acrylic Paint Set, Officeworks) to render some lures black, and give others either white or grey bands (figure 4) to resemble the three colour morphs of *E. annulatus* [17] and also, the putative models for Batesian mimicry (*Laticauda* for the black-and-white bands; *Aipysurus* and *Hydrophis* for the grey bands: see [17]). We used three replicate lures of each colour morph during the trials to avoid stimulus pseudoreplication.

To assess responses of free-living predatory fish, one of us (CG) pulled a lure behind her on a 5-m length of 10 kg monofilament fishing line as she snorkelled a 50-m straight line transect through water 1–3 m deep (figure 4*b*). A second person swam 3 to 5 m behind the lure (depending on water clarity) to score (a) attacks (fish seizes the lure), (b) follows (fish orients to lure and swims behind it, approaching but not seizing the lure) and (c) body lengths of all fishes based on comparison with the 300 mm lure. Follows as well as attacks appear to indicate predatory intent, because (a) all follows were by large (greater than 300 mm long) predatory fishes; and (b) attacks were always preceded by follows. If the colour of the lure affects a fish's willingness to treat it as a prey item, we expect that lures of all colours may attract follows, but that banded lures will be less likely to elicit attacks than will black lures. All data were gathered during daylight hours (1000–1600 h) over a total of 11 days in March 2022. We changed lures between successive trials, in random order but with the restriction that all three colours were tested in each three-trial sequence.

The analysis below does not include responses by small fishes engaged in territorial defence [33,34] rather than predation. These comprised four attacks by small (less than 100 mm) damselfish (*n* = 1 *Pomacentrus chrysurus*, *n* = 3 *Abedefduf whitleyi*) and one attack by a 400-mm graphic tuskfish (*Choerodon graphicus*). Likewise, we disregarded follows by schools of small (50 mm) damselfish (total *n* = 23 individuals of *A. sexfasciatus*).

### (e) Statistical analyses

Using JMP 16.0 and SAS (SAS Institute, Cary, NC), we conducted two sets of logistic regression analyses. The first used trials (one transect with one lure) as the unit of replication, with the result scored conservatively as ‘lure attacked at least once versus not attacked’ and ‘lure followed at least once versus not followed’. This method asks whether the colour of a lure affected whether or not it was either attacked or followed. If a lure was followed then attacked, we scored it only as an attack. The second analysis used individual attacks and follows as the unit of replication, with trial # (set of sequential trials containing each of the three colours of lures) as a random factor to avoid pseudoreplication (conducted using Proc Glimmix in SAS, with a binary distribution and logit link function). This second analysis thus was restricted to trials in which a fish either attacked or followed a lure. The dependent variable was the response of the fish (attack versus follow). We performed this test on the combined dataset, and separately for common fish species. We also conducted multiple logistic regression, by including fish species as well as lure colour (and their interaction) as independent variables and response as the dependent variable.

## 3. Results

### (a) Response of predatory fishes to lures

In a total of 30 trials (10 per lure colour), we recorded 116 follows and 14 attacks by fishes. Overall, lures were approached and/or seized by fishes of 11 species (table 1). Four species attacked the lure, with *Plectropomus leopardus* the most important numerically (table 1).

**Table 1.** Species of predatory fishes that attacked or followed snake-model lures. Table shows mean body lengths and numbers of individual fish that either followed or seized lures.

common name	scientific name	body length (mm)	# follows	# attacks
brown-spotted grouper	*Epinephelus maculata*	400	0	1
blackspot emperor	*Lethrinus harak*	300	3	0
blue trevally	*Caranx melampygus*	400	14	0
brassy trevally	*Caranx papuensis*	500	1	0
camouflage grouper	*Epinephelus polyphekadion*	510	3	1
coral trout	*Plectropomus leopardus*	530	57	10
great barracuda	*Sphyraena barracuda*	600	1	0
giant trevally	*Caranx ignobilis*	750	1	0
long tom	*Tylosurus crocodilus*	590	17	2
yellowtail barracuda	*Sphyraena flavicauda*	310	7	0
yellowtail emperor	*Lethrinus atkinsoni*	410	12	0

**Figure 5.**
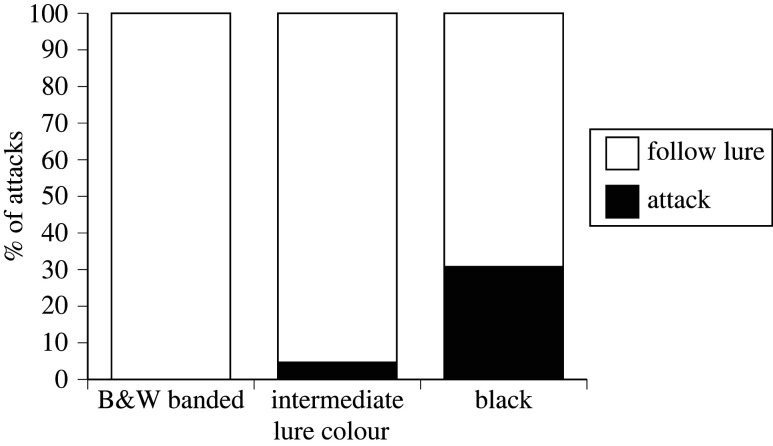
Effect of lure colour on fish response. Figure shows proportion of attacks to lures that were painted to represent three colour morphs of the sea snake *Emydocephalus annulatus*: black-and-white-banded, intermediate (grey-banded) and jet-black (melanic).

The total number of fishes responding (i.e. *n* attacks plus *n* follows) was similar to lures of different colours (*n* = 40 to black, 48 to black-and-white banded, 42 to grey-banded) but almost all attacks were directed to black lures (*n* = 12, versus *n* = 0 to black-and-white and *n* = 2 to grey-banded). Thus, there is no evidence that a lure's colour affected whether or not it induced follows (logistic regression with trial as the replicate, follows in 90% of trials with black-and-white, 80% of trials with grey-banded, 60% of trials with black; *χ*^2^ = 2.63, d.f. = 2, *p* = 0.27) but black lures were six times more likely to be attacked (attacks in six trials with black lures [60% of trials], versus 0 for black-and-white and 2 for grey-banded [20% and 0% of trials]; *χ*^2^ = 11.33, d.f. = 2, *p* < 0.004). Multiple attacks within a single trial were common, and the analysis using individual interactions (follow versus attack) as the dependent variable showed that black lures attracted about 15 times as many attacks (as a proportion of all responses, including follows) than did either of the banded alternatives (*χ*^2^ = 17.02, d.f. = 125, *p* < 0.0002; figure 5).

If we restrict analysis to data from coral trout (*Plectrodromus leopardus*), the fish species responsible for most attacks, the same effect of lure colour was apparent (*n* = 9 attacks and 12 follows to black, *n* = 0 and 31 to black-and-white, *n* = 1 and 14 to grey-banded; *χ*^2^ = 19.15, d.f. = 2, *p* < 0.0001). For all other fish species combined, we similarly recorded more attacks versus follows to black lures (3 attack, 15 follow) than to black-and-white (0 attack, 17 follows) or grey-banded (1 attack, 27 follows) lures. When we included fish species as well as lure colour as independent variables in the logistic regression, the response (attack versus follow) was affected by colour of the lure (*χ*^2^ = 26.02, d.f. = 2, *p* < 0.0001) but did not differ significantly among fish species (*χ*^2^ = 16.59. d.f. = 9, *p* = 0.06) nor by the interaction between lure colour and fish species (*χ*^2^ = 0.29, d.f. = 8, *p* = 1.00).

## 4. Discussion

### (a) Advantages of banded colour morphs

Our study suggests that banded coloration in sea snakes reduces vulnerability to predation. Although snake models have been widely used to explore impacts of colour on rates of attack by terrestrial predators, the immobility of those models and the lack of direct observation of predator-model interactions raises ambiguity about the identity of the predators involved, and whether disturbance to the models may be affected by detectability, predator curiosity or scavenging rather than predation [9]. Also, some colour patterns may affect crypsis only when moving [35]. Our methods overcame those problems by using moving stimuli and directly observing predator–model interactions.

The non-random pattern that we documented (fewer attacks on banded models) cannot be attributed to:

(i) Detectability—banded lures appeared more obvious than black lures to human visual systems but were less likely to be attacked by fishes than were black lures. Lures of all colours may have been obvious to fish (most of which approached the lure from beneath, where it was silhouetted against the sky rather than the darker reef substrate), based on high and similar numbers of follows to lures of different colours. Flicker-fusion can render banded snakes more cryptic in low light levels against complex backgrounds [9,14,15] but in the present study, lures were highly illuminated in shallow clear water. Similar numbers of ‘follows’ to all lure types strongly refute the idea that differences in attack rates were driven by differences in detectability.
(ii) Cue novelty—fish might exhibit neophobia, avoiding novel stimuli, but snakes of all three colour morphs occur in both of our study sites. Black snakes are more common than banded snakes in the Baie des Citrons study area, but banded snakes (*Laticauda* spp.) dominate in the Ile aux Canards site (R.S. & C.G. 2021, unpublished data). The relative numbers of attacks to black versus banded lures did not differ significantly between the two study sites (*χ*^2^ = 2.04, d.f. = 2, *p* = 0.36).
(iii) Curiosity—the fishes vigorously seized the moving models rather than investigating them cautiously (figure 4*c*), and the only fish to exhibit follows or attacks were large individuals of predatory species. If curiosity stimulated responses, we would expect approaches by the far more abundant small and non-piscivorous fish taxa.
(iv) Scavenging—the lures were moving rapidly and directionally through the water when seized by predatory fish, inconsistent with dead animals.

We do not know why fishes often followed lures without seizing them, but similar responses to artificial lures are usually attributed to fish evaluating multiple traits of the stimulus before launching an attack [36]. In our study, the six species that followed the lure but never attacked it included the four smallest-bodied taxa (*Caranx melampygus*, *Lethrinus atkinsoni*, *L. harak*, *Sphyraena flavicauda*) but also the two largest (*Caranx ignobilis*, *Sphyraena barracuda*), so predator body size did not have any simple relationship to rates of attack. Predatory fish can use chemosensory cues to identify potential prey items [37], but scent cues from our lures would have been minimal. Because colour was the only variable differentiating the three types of lures, we conclude that large predatory coral-reef fish evaluate colour as a relevant stimulus when they encounter a snake-shaped object moving through the water.

The reluctance of predatory fishes to attack banded lures might reflect innate avoidance or learning; both of these mechanisms have been demonstrated in avian predators [38], and are plausible in fishes also [39,40]. The hypothesis of learned avoidance of deadly snakes has been criticized on the grounds that encounters are likely to be fatal for predators [9], but the small heads and short fangs of many sea snake species [24] may increase the likelihood of non-fatal interactions. Both of these mechanisms—learning and innate avoidance—rely upon the ability of predatory fish to distinguish between uniformly dark versus banded patterns. That assumption is plausible, because the primary difference between banded and black lures—the contrasting bands—provide sharp edges that facilitate recognition and avoidance learning in marine fishes [39]. Concentric rings of contrasting colours maximize stimulation across the retina and increase overall salience of the signal [3,41].

In combination, available data are consistent with the hypothesis that long-term stability of colour polymorphism in *Emydocephalus annulatus* is maintained by frequency-dependent predation [17] and advantages in pollutant excretion [31]. That is, snakes in our study areas benefit from melanism because it enhances their ability to eliminate trace elements when they slough their skins; but melanic individuals experience a higher risk of predation. By contrast, banded morphs void fewer pollutants, but are protected from predation by their resemblance to deadly snakes. The grey-banded morph of *E. annulatus* may derive both benefits because banding early in life protects it from predation when it is most vulnerable; and ontogenetic darkening confers the pollutant-excretion advantage of melanism. Many species of snakes both on land and in the ocean exhibit brighter bands when young [28,42]. In sites that are not polluted, the pollutant-excretion advantages of melanism are reduced whereas the benefits of banding are higher (deadly models and large predators are more abundant), consistent with the observation that most *E. annulatus* from unpolluted sites are banded [31].

Our data also support suggestions that venomous sea snakes have been models for the evolution of banding mimicry in eels [43], sharks [44], holothurians [45] and octopuses [46]. Avoidance of banded patterns by predators is a core assumption of all of these suggestions. Mullerian mimicry may be involved as well, because the banded coloration of most marine snakes may provide a common signature of toxicity that enhances protection against predators [24,43] (but see [45]). An alternative possibility is innate avoidance of banded patterns by predatory fish, driving convergent evolution of such patterns in many types of marine organisms.

Geographical similarity in assemblages of predatory fish and snakes across the IndoPacific, at least at the generic level, suggests that our results may apply broadly. Similar studies in coral-reef systems where sea snakes do not occur (e.g. French Polynesia) could examine the responses of predatory fish to colour patterns of prey in the absence of selection to avoid dangerously venomous snakes. Such a study could address the alternative hypothesis of innate avoidance of banded patterns rather than mimicry of venomous snakes (above). Models that manipulated colours in ways that do not mimic snake patterns (e.g. dots rather than bands) could further test the possibility of innate avoidance to specific patterns. Future work also could extend our methods to terrestrial systems, by utilizing moving models (perhaps robotic) to reduce ambiguity about the identity and motivation of predators. Importantly, the use of models overcomes many of the ethical objections to studies using live prey items.

## Data Availability

Data have been deposited in the Dryad Digital Repository: https://doi.org/10.5061/dryad.dv41ns227 [47].
